# Efficacy of Memantine in Schizophrenic Patients: A Systematic Review

**DOI:** 10.1155/2017/7021071

**Published:** 2017-01-24

**Authors:** Giuseppe Di Iorio, Gaia Baroni, Marco Lorusso, Chiara Montemitro, Maria Chiara Spano, Massimo di Giannantonio

**Affiliations:** ^1^Department of Neuroscience Imaging and Clinical Science, “G. d'Annunzio” University of Chieti, Chieti, Italy; ^2^Department of Mental Health, National Health Trust, Chieti, Italy

## Abstract

Several evidences support the hypothesis that glutamatergic dysfunction may be implicated in the pathogenesis of schizophrenia and in the last few years great interest has been focused on the role of the N-methyl-D-aspartate receptor (NMDAR). Glutamate is the main excitatory neurotransmitter in human CNS and it plays a prominent role in synaptic plasticity, learning, and memory and other cognitive functions. Increasing interest in memantine add-on therapy in schizophrenic patients with negative and cognitive symptoms may suggest that memantine could be a new promising treatment in schizophrenia. The aim of this update was to evaluate clinical data about the memantine effectiveness in schizophrenic patients. Our systematic review of the literature highlights that memantine therapy in schizophrenic patients seems to improve mainly negative symptoms while positive symptoms and cognitive symptoms did not improve significantly.

## 1. Introduction

Schizophrenia is a chronic illness characterized by positive symptoms and negative symptoms (such as reduction of emotional responsiveness, motivation, socialization, speech, and movement) that leads to progressive functional and cognitive impairment.

The aetiology of schizophrenia is not well determined, but alterations and disturbances of developmental processes have been hypothesized by many researchers [1, 2].

For many years, a glutamatergic dysfunction has been implicated in the pathogenesis of schizophrenia [3] and recently great interest has been focused on the role of the N-methyl-D-aspartate receptor (NMDAR) [4, 5]. Furthermore, a recent meta-analysis indicates that schizophrenia is associated with glutamate level increase in several brain regions [6]. Glutamate is the main excitatory neurotransmitter in human CNS and it plays a prominent role in synaptic plasticity, learning, and memory and other cognitive functions [7]. It has been also involved in excitotoxicity and neuroprotection processes [8]. Glutamate predominant biological use is not as a neurotransmitter but as an amino acid building block of protein synthesis [9]. NMDAR is a glutamate ionotropic receptor (such as AMPA and kainate receptors) and it was identified 30 years ago because of the selective antagonism by D-2-amino-5-phosphonovaleric acid [10]. NMDARs are voltage-dependent channels, they show high permeability to Ca^2+^ and could be blocked by endogenous Mg^2+^ [11]. Under physiological condition, NMDARs are activated, allowing calcium ion flux into the cell, and suddenly blocked by Mg^2+^ [12]. In some chronic neurodegenerative disorders, NMDARs could be overactivated. An excessive Ca^2+^ flux into the nerve cell due to pathologically activated NMDARs leads to cellular oxidative injury and cell-death: this process is known as excitotoxicity. Glutamate-mediated excitotoxicity is due to an excessive stimulation of glutamate receptors even if extracellular glutamate levels are normal [12]. Pathogenesis of Alzheimer's disease has already been linked to excitotoxicity [13] and recently many studies have explored a hypothetical role of excitotoxicity in schizophrenia brain abnormalities [14, 15]. The link between glutamate, NMDAR, and schizophrenia was firstly suggested by psychotomimetic symptoms induced by NMDAR noncompetitive antagonists, such as ketamine and phencyclidine, in healthy subjects [16]. These substances bind the intrachannel site of the receptor and prevent calcium ion flux into the cell, leading to effects that mimic those seen in schizophrenia including hallucinations, delusions, thought disorder, and, most notably, negative symptoms [16–18]. Ketamine and PCP were also shown to lead to a relapse of psychotic symptoms in previously stabilized patients with schizophrenia, worsening negative, and cognitive symptoms [5]. Furthermore, it has been observed that repeated subcutaneous injections of NMDA channel blockers caused neurodegenerative changes in rat cortex which coincided with structural changes seen in schizophrenia [17]. All compounds binding the PCP site on NMDAR seem to induce psychosis, and the same is true for antagonist at both glutamate binding site and glycine modulatory site [19]. Another supporting evidence to NMDAR role in psychosis has been provided by autoimmune disorders of the CNS: NMDA receptor antibodies may conduce to psychotic symptoms, such as anxiety, agitation, insomnia, aggression, visual or auditory hallucinations, paranoia, grandiose delusions, hyperreligiosity, sexual disinhibition, mania, psychosis, or catatonia [20, 21]. Among NMDAR antagonist compounds, recently, great emphasis has been placed on memantine, a derivative of amantadine, an anti-influenza agent. Memantine is a noncompetitive NMDARs antagonist. It is defined as an “open-channel blocker” and, at the same time, a “trapping channel blocker” of NMDARs. In fact, memantine can enter the channel and block current flow only if the channel is open; then, when it enters the open-channel of NMDARs and agonists unbind it, memantine is trapped inside the channel [11]. An important observation is that memantine binds the same site of NMDARs of Mg^2+^, endogenous blocker of NMDARs [11], with moderate affinity and rapid unblocking kinetics [22]. Also ketamine and phencyclidine (PCP) are “open-channel blockers” of NMDARs but they show slower unblocking kinetics and less voltage- and use-dependency in comparison with memantine and Mg^2+^ [23]. Memantine is the most readily reversible open-channel blocker and it shows a strong functional voltage-dependency [23]: these properties allow memantine to not affect the physiological activation of NMDARs whereas it blocks the sustained activation under pathological conditions [23]. Under resting conditions, all “open-channel blockers” occupy the NMDA receptor channel. Both Mg^2+^ and memantine are voltage-dependent antagonist and are able to leave the NMDA receptor channel upon strong synaptic depolarization. Instead, the slower blockers, as ketamine and PCP, remain trapped in the channel [23]. We could say that memantine can recognize and block pathologically activated NMDARs whereas it does not influence the normal functioning of physiologically activated receptors, and this property is not shared by ketamine and PCP: this could be the reason why although ketamine, PCP, and memantine are all NMDARs antagonist, they show so different effects.

Memantine was first synthesized by Eli Lilly and Co. and patented in 1968. Nowadays, according to its neuroprotective properties [13] and with the “glutamate hypothesis” of Alzheimer's disease [24], memantine is recommended for treatment of moderate Alzheimer's disease in people who do not tolerate AChE inhibitors and for management of severe Alzheimer's disease [25]. In fact, AD is defined by neurodegenerative condition linked with the presence of *β* amyloid (A*β*) tau neurofibrillary tangles [26, 27], predominantly in the hippocampus. According to evidence, the injurious effects of amyloid *β* peptide (A*β*) in Alzheimer's disease (AD) may be mediated by excessive activation of NMDARs: soluble oligomers of A*β* are thought to mimic extracellular glutamate stimulation of NMDARs and disrupt synaptic plasticity and long-term potentiation, eventually leading to synaptic loss and causing dementia [28, 29].

Finally preclinical studies have demonstrated that memantine at high concentrations targets many receptors, including serotonin receptors, nicotinic acetylcholine receptors, sigma-1 receptors, and serotonin and dopamine [11].

In view of the important role of memantine in glutamatergic dysfunction in Alzheimer's disease and in the light of the involvement of glutamatergic system both in Alzheimer and in schizophrenia, we wondered if this treatment should be considered as a valid option for negative and cognitive symptoms in schizophrenia.

Our aim was to evaluate clinical data about the memantine effectiveness in schizophrenic patients, as it may represent a valid option of treatment for negative and cognitive symptoms of schizophrenia; with this purpose we conducted a systematic review of the literature.

## 2. Methods

We searched on PubMed to identify original studies about the use of memantine in treatment of schizophrenic patients. The following search words were used: “memantine” AND (“psychosis OR schizophrenia”). The search was conducted on June 16, 2016, and yielded 135 records. Inclusion criteria were original articles (open label or double-blind trials, prospective or retrospective observational studies, and case reports) written in English, patients' age ≥18 years, patients affected by schizophrenia, and treatment with memantine. Animal studies, reviews, commentaries, letters to the editor, studies enrolling patients with organic comorbidity, and studies not enrolling schizophrenic patients or not including a treatment with memantine were excluded.

**Figure 1:**
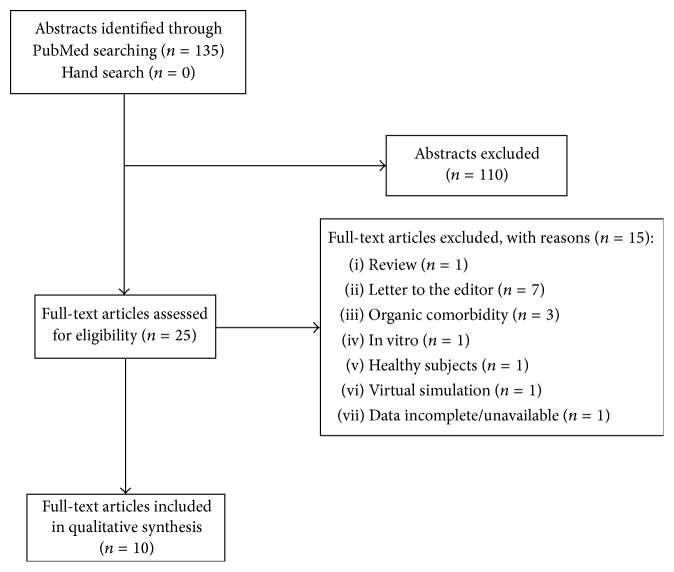
Flowchart of the systematic review.

All the authors agreed on inclusion and exclusion criteria. We excluded 110 records by reading titles and abstracts. By reading the full texts of the 25 remaining articles, we found 10 papers meeting our inclusion/exclusion criteria and therefore were included in the qualitative synthesis (Figure 1).

## 3. Results

### 3.1. Memantine and Positive Symptoms

Only one study supports the efficacy of memantine combined with antipsychotic therapy in management of positive symptoms: in a 6-week clinical trial of 2008, 7 patients were treated with antipsychotics and memantine 20 mg/day. Between entry and endpoint scores, BPRS decreased at 18%, CGI decreased at 19%, and PANSS positive subscale score decreased at 15.6% [30]. On the other side, in a 12-week double-blind, placebo-controlled trial conducted on 26 subjects mean PANSS scores were not different between the memantine and placebo groups [31].

### 3.2. Memantine and Negative Symptoms

Memantine effects on negative symptoms have been evaluated by numerous studies. Two case reports showed an effective improvement in negative symptoms when memantine 10 mg/day was added to conventional therapies [32]. In a retrospective study based on 26-patient case series a satisfactory therapeutic response was obtained with antipsychotic drugs and 20 mg/day of memantine [33]. Krivoy et al. observed a 16% reduction in total PANSS and a 21% reduction in PANSS negative subscale [30]. In 2015, a 26-week randomized double-blind, placebo-controlled crossover study conducted on 52 patients showed that PANSS negative symptoms scores significantly improved in subjects treated with clozapine and memantine 10 mg/day compared to those treated with placebo [34]. A 12-week randomized controlled trial conducted on 60 subjects showed higher scores of QLS (quality of life scale) and GAF (global assessment of functioning) in patients treated with memantine compared to the control group [35]. In 8-week double-center, randomized, double-blind, placebo-controlled, parallel-group study conducted on 40 subjects, patients taking memantine and risperidone showed PANSS total and PANSS negative subscale scores lower than those treated with placebo [36]. Finally, a multicenter 8-week double-blind, randomized, placebo-controlled study, conducted on 138 patients did not achieve statistically significant results [37].

### 3.3. Memantine and Cognitive Symptoms

Cognitive effects of memantine have been investigated in few studies. Veerman et al. found that composite memory score significantly improved during memantine add-on of clozapine therapy compared to placebo in resistant schizophrenic patients [34].

Krivoy et al. also evaluated neurocognitive aspects of patients taking memantine but NCSE did not show any significant change before and after therapy, as well as scores for CDT (clock drawing test) [30]. MMSE (Mini Mental State Examination) was performed in some studies without statistically significant results [31, 32].

Swerdlow et al. evaluated in 41 chronic psychotic patients and 43 healthy subjects the sensorimotor gating function measured by prepulse inhibition (PPI) and mismatch negativity (MMN) [38]. Those functions are impaired in patients with chronic psychotic disorder. According to their results, 20 mg of memantine enhanced PPI and MMN performances due to its function on neuroplasticity [38]. Memantine produced dose-dependent increases in measures of preattentive functions [38].

### 3.4. Memantine and Side Effects

Conflicting evidences exist about memantine side effects. On the one hand, several studies have not documented important side effects [30–33, 35, 36]; on the other hand, some studies have reported some. Some patients complained of headache, insomnia, constipation, fatigue, dizziness, and auditory hallucinations [34, 37]. Veerman et al. found an increase on the Allergic Reactions subscale of the LUNSERS, including rash, sensitivity to sun, unusual skin marks, and itchy skin [34]. Considering metabolic effects of memantine, a case report has described a loss of weight due to a combined treatment with clozapine and memantine [39].

## 4. Discussion

In this review we selected trials and reports of patients treated with memantine in order to improve cognitive impairment and symptoms in schizophrenic patients.

It seems that negative symptoms improved in the large majority of patients treated. However, according to results summarized in Table 1, there is no clear evidence of memantine's action in improving cognitive performances, as many factors may play an unknown role. We could hypothesize that some medications, such as mood stabilizers, antidepressants, or antipsychotics, may interfere with memantine's actions on NMDA receptors. In fact, even though all trials excluded patients with addictions, it was impossible to exclude patient treated with other drugs, while all healthy control subjects were drug naïve.

2 of the 10 studies we finally selected were case reports, obviously characterized by a small sample size and/or short follow-up.

Many studies differ on tests used to monitor clinical response to memantine although the majority of the trials we found used PANSS score to evaluate positive and negative symptoms and MMSE to evaluate cognitive impairment.

Only Swerdlow et al. tested cortical response to stimuli in patients with chronic psychotic disorders and noticed a dose-dependent improvement in PPI and MMN performances in patients treated with 20 mg of memantine [38].

We excluded letters to editor mainly because data were incomplete. Anyway we consider that it is useful to report some of them because of their originality. In fact, only one trial [40] used fMRI combined to working memory tests but only 7 patients were enrolled (among them only 3 took memantine and 4 took placebo) compared to 7 controls. Another fMRI study conducted on healthy subjects showed significant reduction in activation in prefrontal cortex and anterior cingulate cortex after 10 mg memantine daily administration for 21 days, compared to the drug naïve session [41]. These are very interesting pilot studies but further trials with a more numerous sample are required to obtain an objective result. Moreover, it is not clear which dose of memantine should be administrated to obtain the best result. In fact, some evidence suggests that 10–15 mg/kg of memantine may minimize schizophrenia-like symptoms in ketamine-induced model in rats [42] while 20 mg/kg of memantine seems to impair rather than ameliorate cognitive function [43, 44] but memantine (5 mg/kg) did not increase Homer1a signal [22].

Favourable effects of memantine addition to nonclozapine antipsychotics have been described by a consistent number of studies. However, memantine is considered more effective as an adjunctive therapy to clozapine than to nonclozapine antipsychotics [45].

In addition to the study conducted by Veerman et al. mentioned in results [34], De Lucena et al. reported, in a letter to editor, the efficacy of memantine as add-on therapy to clozapine with large effect sizes for overall symptoms, positive symptoms, negative symptoms, and global cognitive functioning in patients with partial remission of negative symptoms of schizophrenia [46]. In the same study it has been further hypothesized that BDNF may play a role in the effect of memantine in patients with schizophrenia, but inconsistent results have been found [46]. The positive effects of memantine augmentation to clozapine may be linked to their conjunct action on NMDA receptors. This combination modulates glutamatergic neurotransmission at multiple levels [45], with clozapine inducing both upregulation of *α*-amino-3-hydroxy-5-methyl-4-isoxazolepropionic acid (AMPA) receptors and NMDA receptors [34]. Schaefer et al. 2007 described a memantine-mediated decrease of clozapine-induced weight gain using an on-off-on design with a significant increase of weight after discontinuation and again a substantial weight loss after reexposition with memantine [39]. In fact, some studies suggest that glutamatergic mechanisms play a role in weight regulation and eating behaviour [47]. Furthermore, memantine has been shown to decrease weight in obese women with binge-eating disorder [48].

According to De Bartolomeis et al., ketamine, MK-801, and memantine induce different changes in Postsynaptic Density (PSD) protein transcripts, despite their apparently similar pharmacological action. Homer1b and PSD-95 are key PSD molecules involved in synaptic rearrangements underlying dendritic spine growth and synaptic strength [7, 49], and the downregulation of Homer1b has been found to attenuate glutamate-mediated excitotoxicity in rat cortical neurons [50]. Furthermore, recent studies explain how excitotoxicity appears strongly linked to a reduction of BDNF, which plays an important role in survival of striatal neurons. In fact, in excitotoxicity, excess influx of Ca^2+^ through NMDARs activates CREB shut-off pathway, which blocks BDNF expression leading to neuronal death [51]. We believe that further studies on BDNF correlation with memantine treatment are required, given that both are involved in neuroplasticity processes and may represent the key to explain memantine's action.

In conclusion, memantine therapy in schizophrenic patients has given unclear results. It seems that memantine improves mainly negative symptoms while cognitive and positive symptoms did not improve significantly. As already suggested by De Bartolomeis et al., memantine may improve clinical outcomes, especially when administered as add-on therapy of clozapine [7]. We believe that use of memantine should be considered in patients with prevalent negative symptoms and cognitive impairment, even if further trials are required. Memantine could be a new opportunity to treat young patients in order to prevent further cognitive decline that will lead to global impairment.

## Figures and Tables

**Table 1:** Observation during memantine administration.

Articles included	Positive symptoms	Negative symptoms	Cognitive symptoms	Side effects
Krivoy et al. 2008	↓	↓	×	—
Lee et al. 2012	×	×	×	—
Paraschakis 2014	×	↓	×	—
John et al. 2014	×	↓	×	—
Veerman et al. 2016	×	↓	↓	+
Omranifard et al. 2015	×	↓	×	—
Rezaei et al. 2014	×	↓	×	—
Lieberman et al. 2009	×	×	×	+
Schaefer et al. 2007	×	×	×	—
Swerdlow et al. 2016	×	×	↓	—

↓ reduction; × not evaluated/no variation; + reported; — not evaluated/not reported.
